# Gender Differences in the Association between Physical Inactivity and Mental-Health Conditions in People with Vision or Hearing Impairment

**DOI:** 10.3390/ijerph19063307

**Published:** 2022-03-11

**Authors:** Guillermo F. López-Sánchez, Lee Smith, Rubén López-Bueno, Shahina Pardhan

**Affiliations:** 1 Department of Public Health Sciences, Division of Preventive Medicine and Public Health, School of Medicine, University of Murcia, Espinardo, 30100 Murcia, Spain; gfls@um.es; 2 Centre for Health, Performance and Wellbeing, Anglia Ruskin University, Cambridge CB1 1PT, UK; shahina.pardhan@aru.ac.uk; 3 Department of Physical Medicine and Nursing, University of Zaragoza, 50009 Zaragoza, Spain; rlopezbu@unizar.es; 4 Faculty of Health, Vision and Eye Research Institute, School of Medicine, Education, Medicine and Social Care, Cambridge Campus, Anglia Ruskin University, Cambridge CB1 1PT, UK

**Keywords:** physical activity, mental health, visual impairment, hearing impairment

## Abstract

This study aimed to examine associations between physical inactivity and mental health in Spanish adults with vision or hearing difficulties and explored differences between men and women. Data from the Spanish National Health Survey in 2017 were analyzed (*n* = 23,089 adults, 15–103 years, mean age 53.4 ± 18.9 years, 45.9% men). Physical inactivity (exposure) was evaluated with the International Physical Activity Questionnaire Short Form. Participants self-reported whether they had previously suffered from depression, chronic anxiety and other mental-health complications (outcomes). Associations between physical inactivity and mental-health complications were assessed with multivariable logistic regression in people with difficulty seeing and hearing, after grouping by gender and adjusting for age, body-mass index, education level, living as a couple, smoking and alcohol consumption. The overall multivariable logistic-regression analyses showed that women with vision impairment showed significant associations between physical inactivity and depression (OR 1.403, 95% CI 1.015–1.940) and other mental-health complications (OR 2.959, 95% CI 1.434–6.104). In the overall analyses, there were no significant associations in men and in people with hearing impairment. The age-stratified analyses showed that inactive people with visual impairment who were <65 years old had a higher risk of mental-health conditions. In conclusion, physical activity has been shown to be important in the prevention of mental-health complications. Healthcare practitioners and policy makers should look at appropriate strategies to increase levels of physical activity in people with vision loss, especially in women and in those <65 years old.

## 1. Introduction

Mental-health complications pose significant global quality-of-life challenges, with anxiety and depression being the most prevalent mental-health complications [1,2]. Depression has been estimated to affect approximately 264 million people worldwide [1]. Moreover, it is one of the main global causes of disability, considerably contributing to the global burden of disease [1]. Anxiety has been estimated to affect approximately 284 million people worldwide [2].

It is important to identify population groups with a high prevalence of mental-health complications in order to reduce their prevalence through targeted prevention efforts. It is known that people with low levels of physical activity show a higher risk of depression and anxiety [3,4,5,6]. It is also known that people with disability (e.g., vision or hearing impairment) also show higher risk of depression [7], cognitive impairment, dementia [8,9] and perceived discrimination [10]. In addition, it is known that women have a higher risk of mental-health conditions [11].

What is not known is the association between physical activity and mental health complications among those with vision or hearing difficulties and specifically when stratified by gender. Such analyses are important to identify those at greatest risk of mental-health conditions in order to inform targeted interventions. To our knowledge, no study has explored gender differences with regard to the association between physical inactivity (i.e., not meeting physical-activity recommendations) and mental-health complications in people with vision or hearing impairment. This is particularly important because people with vision or hearing impairment are an especially vulnerable group and they face numerous barriers to accessing physical activity [12,13], and gender differences could play a fundamental role in this access to physical activity. The aim of this study was to investigate cross-sectional gender-specific associations between physical inactivity and mental-health complications in representative samples of Spanish people with vision or hearing impairment. We hypothesize that associations between physical inactivity and mental-health complications will be significant in people with vision or hearing impairment, considering our previous research [11,14]. 

## 2. Methods

### 2.1. The Survey

**Figure A1:**
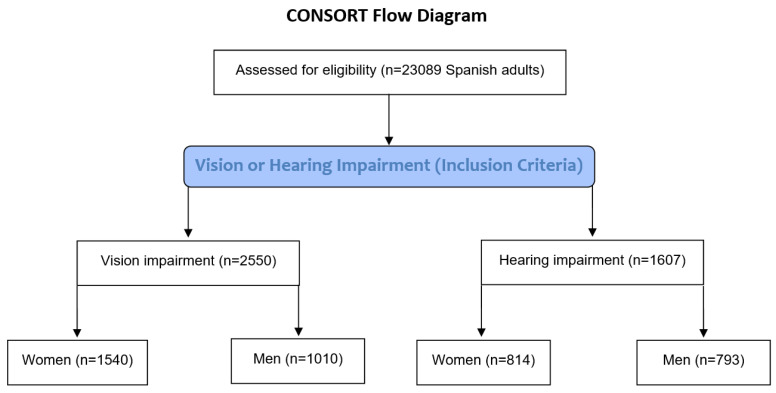
CONSORT Flow Diagram.

The present study utilized data from the Spanish National Health Survey (year 2017) with data collection taking place between October 2016 and October 2017. Therefore, the design of this study was cross-sectional. A detailed description of the Spanish National Health survey is provided in the previous literature and here we provide a brief overview [15,16]. A three-stage stratified sampling method was employed for data collection. In the first stage, census sections were taken into consideration, in the second stage the family dwellings, and in the third stage an adult (older than 15 years) was chosen within each dwelling. To select the sections in each stratum, the probability was proportional to their size. Regarding the dwellings, they were chosen with equal probability in each section using systematic sampling and prior arrangement considering the dwelling size. Therefore, due to this process, samples were self-weighted in each stratum. In order to select the person to answer the adult questionnaire, the random Kish method was applied, which assigns the same probability to all adults older than 15 years living in the household. As is illustrated in Figure A1 (Appendix A), a total of 23,089 adults (age range: 15–103 years) participated in this survey, allowing for a representative sample of the adult Spanish population, of which 2550 people (1540 women and 1010 men) had vision impairment alone and 1607 people (814 women and 793 men) had hearing impairment alone. The CAPI (computer-assisted personal interviewing) method was used for the data collection, and it was implemented in the households of the participants. The interviewers who completed the questionnaires with the responses of the participants had been previously trained in this method. An informed-consent form was signed by all participants before responding to the questionnaire. This research was conducted in accordance with the Declaration of Helsinki of the World Medical Association. In accordance with the regulation of the European Union, the file data for public use does not require the approval of an accredited ethics committee for statistical or research purposes.

### 2.2. Vision or Hearing Impairment (Inclusion Criteria)

Participants who answered affirmatively to the question ‘‘Do you have difficulty seeing?” were considered to have vision impairment (some difficulty/much difficulty/blind). This group included participants who may not be habitually using their spectacles or contact lenses, and also those who reported difficulty in seeing with their own spectacles/contact lenses. 

Those who answered affirmatively to the question ‘‘Do you have difficulty hearing what is being said in a conversation with another person in a quiet place?” were included in the ‘hearing impairment’ group (some difficulty/much difficulty/deaf). This group was composed of people who may not be using a hearing aid and also those who had difficulty hearing with their hearing aid.

These questions are part of the Spanish National Health Survey (year 2017). The questions belong to the Budapest Initiative Task Force on Measurement of Health Status [17], and the International Classification of Functioning, Disability and Health [18] was adopted.

### 2.3. Physical Inactivity (Exposure)

The International Physical Activity Questionnaire (IPAQ) Short Form was used to measure the exposure variable of physical activity. The unit of physical activity used was MET-minutes/week, where MET is the Metabolic Equivalent of Task. Total physical activity MET-minutes/week were calculated through the following formula: sum of walking + moderate + vigorous MET-minutes/week scores [19]. Participants were divided into two categories according to the guidelines for data processing and analysis of the IPAQ [19]: (1) fewer than 600 MET-minutes/week and (2) at least 600 MET-minutes/week, the latter of which is equivalent to meeting current physical-activity recommendations. Adults aged ≥70 years did not complete the IPAQ Short Form, as this questionnaire was developed for population surveillance of physical activity among adults aged 15−69 years, and its use with older and younger age groups is not recommended [19]. The IPAQ has been validated in adult populations from different countries, showing acceptable validity (ρ = 0.30, 95% CI: 0.23–0.36) and reliability (Spearman’s ρ = 0.81, 95% CI: 0.79–0.82) [20]. Specifically, the IPAQ Short Form has been validated among Spanish university students, showing adequate validity [21]. 

### 2.4. Mental-Health Complications (Outcome)

Mental health was evaluated through the following yes/no questions: 1. “Have you ever been diagnosed with depression?”; 2. “Have you ever been diagnosed with chronic anxiety?”. 3. “Have you ever been diagnosed with other mental problems?”. Those who answered affirmatively were considered to have depression/chronic anxiety/other mental problems. These questions of the Spanish Health Survey 2017 have been previously validated [22] and widely used in the previous scientific literature [6,23,24,25,26].

### 2.5. Gender and Other Covariates

Participants self-reported their gender (woman or man) and all analyses were carried out following the international recommendations on gender in public-health research [27,28,29,30]. The selection of the other control variables was based on the past literature [31,32,33,34]. Sociodemographic variables included age, education level and living as a couple. Education was based on the highest educational level achieved and was categorized as ≤primary, secondary, and ≥tertiary. Living as a couple was categorized as yes/no. Smoking status was self-reported and categorized as never, current smoker, and past smoker. Alcohol consumption in the last 12 months was self-reported and categorized as yes (any) and no (none). Height and weight were self-reported, and body-mass index (BMI) was calculated as weight in kilograms divided by height in meters squared. 

### 2.6. Statistical Analysis

The statistical analysis was performed with SPSS 23.0 (IBM, Armonk, NY, USA). The prevalence of physical inactivity in people with vision or hearing impairment was calculated by gender (men and women) and by mental-health conditions (depression, chronic anxiety, and other mental problems). These differences were initially assessed by the chi-squared test, providing the frequencies and percentages of each group and their significant differences. In addition, the effect size was calculated using Cohen’s *d*, which is classified as small (0.20), medium (0.50) and large (0.80) [35]. We then conducted a multivariable logistic-regression analysis to assess the association between physical inactivity (exposure) and mental-health conditions (outcome) in people with vision or hearing impairment, stratified by gender. The models were adjusted for age, BMI, education level, living as a couple, smoking and alcohol consumption, as these are the variables that have been shown in the literature as important covariates [11,14,36,37,38,39,40]. All the variables were included in the models as categorical variables with the exceptions of age and BMI, which were included as continuous variables. Results from the logistic-regression analyses are presented as odds ratios (ORs) with 95% confidence intervals (CIs). A complete-case analysis was carried out. Details on the missing data from those with visual impairment were as follows: physical inactivity (*n* = 802; 31.5%), depression (*n* = 0; 0%), chronic anxiety (*n* = 0; 0%), other mental problems (*n* = 0; 0%), age (*n* = 0; 0%), BMI (*n* = 178; 7.0%), education level (*n* = 0; 0%), living as a couple (*n* = 13; 0.5%), smoking (*n* = 5; 0.2%), alcohol consumption (*n* = 4; 0.2%). Details on the missing data from those with hearing impairment were as follows: physical inactivity (*n* = 927; 57.7%), depression (*n* = 0; 0%), chronic anxiety (*n* = 0; 0%), other mental problems (*n* = 0; 0%), age (*n* = 0; 0%), BMI (*n* = 107; 6.7%), education level (*n* = 0; 0%), living as a couple (*n* = 3; 0.2%), smoking (*n* = 1; 0.1%), alcohol consumption (*n* = 1; 0.1%). The level of statistical significance was set at *p* < 0.05.

## 3. Results

Table 1 provides the data on the physical inactivity in people with vision or hearing impairment, by gender and by mental-health condition. Of the 23,089 total adults, there were 2550 people with vision impairment alone and 1607 people with hearing impairment alone. In the group with vision impairment, physical inactivity was significantly more prevalent in women with depression (44.4%) than in women without depression (34.9%), in men with chronic anxiety (45.0%) than in men without chronic anxiety (33.4%), and in women with other mental problems (66.7%) than in women without other mental problems (35.9%). In the group with hearing impairment, physical inactivity was significantly more prevalent in women with depression (55.1%) than in women without depression (41.6%).

The multivariable logistic-regression analyses (Table 2) demonstrate that women (but not men) with vision impairment showed significant associations between physical inactivity and depression (OR 1.403, 95% CI 1.015–1.940) and between physical inactivity and other mental problems (OR 2.959, 95% CI 1.434–6.104). There were no significant associations between physical inactivity and mental-health conditions in the group with hearing impairment. (Table 2). For more details, please observe the Table A1, Table A2 and Table A3 in Appendix B, in which we present all the associations between the covariates of the models (age, BMI, education level, living as a couple, smoking and alcohol consumption) and the outcome variables depression (Table A1), chronic anxiety (Table A2) and other mental problems (Table A3).

In addition, the results stratified by age are presented in Table A4 (Appendix B). Age stratification showed that the association between physical inactivity and mental-health conditions was significant only in those with vision impairment who were <65 years old. In this group, there was a higher risk of depression in inactive women (OR 1.429, 95% CI 1.010–2.021), a higher risk of chronic anxiety in inactive men (OR 1.753, 95% CI 1.037–2.963), and a higher risk of other mental problems in both inactive women (OR 3.101, 95% CI 1.416–6.791) and inactive men (OR 4.287, 95% CI 1.267–14.510).

## 4. Discussion

### 4.1. Main Findings

The overall adjusted multivariate analyses in this study showed significant associations between physical inactivity and mental-health complications (depression and other mental problems) in women with vision impairment. The overall analyses did not show significant associations in men or in either gender group with hearing impairment.

However, in the age-stratified analyses, some significant associations also appeared in men. In brief, the age-stratified analyses showed that inactive women with vision impairment who were <65 years old had a higher risk of depression and other mental problems, while inactive men with vision impairment who were <65 years old had a higher risk of chronic anxiety and other mental problems.

### 4.2. Interpretation of the Findings

Various reasons could explain these results in the overall adjusted multivariate analyses. First, physical inactivity is associated with a worse body image and a lower body satisfaction [41,42], and previous literature has reported that women tend to give more importance to body image than men [41,42]. It is possible that the association between physical inactivity and mental-health conditions was significant in women with vision impairment because they were probably the study group with the worse body image (not available in this survey), and physical inactivity can worsen the problem of body dissatisfaction, thereby increasing the risk of depression and other mental problems in that population group. Previous research [43] has shown that body-image dissatisfaction in visually impaired women is a very important problem, being especially complicated the case of congenitally blind women because they have never been able to see their own image or hold an internal representation, albeit impoverished and systematically distorted, as the tactile kinesthetic information does not fully compensate for the visual experience in the formation of the representation [44]. Another possible factor that may explain the results found in the current study refers to social inclusion. Women with visual impairment may have higher problems with social exclusion due to the emotional and psychological consequences of sight loss [11,36,45], thereby discouraging them from taking part in physical activity. Physical activity can lead to social inclusion [46], and if women with visual impairment cannot carry out physical activity, this may lead to social exclusion, which is in turn associated with mental-health conditions [47]. Additionally, another possible mediator is the gender differences in terms self-confidence in physical activity, as self-confidence in physical activity by women has been an important concern of some researchers in sport psychology [48].

Regarding the results in the age-stratified, adjusted multivariate analyses, it is possible that the higher risk of chronic anxiety and other mental problems found in inactive men with vision impairment who were <65 years old is explained by this population group having sedentary and stressful jobs, with increased stress due to their visual impairment. Therefore, in this specific group, physical activity could be a very useful tool to reduce work stress, chronic anxiety and other mental problems [26,49,50].

Another important factor that can play an important role in the gender differences in the association between physical inactivity and mental-health conditions among people with vision or hearing impairment refers to addictions, such as tobacco addiction or alcoholism [51]. In fact, our results showed that the covariates of smoking and alcohol use significantly predicted mental-health conditions in several of the population-group studies (Table A1, Table A2 and Table A3). Smoking was associated with a higher risk of depression and chronic anxiety in women and men with vision impairment (Table A1 and Table A2). Alcohol was associated with a lower risk of depression in women and men with vision impairment and in men with hearing impairment (Table A1), with a lower risk of chronic anxiety in men with vision or hearing impairment (Table A2), and with a lower risk of other mental problems in women and men with vision impairment and in men with hearing impairment (Table A3). The detrimental effect of smoking for mental-health conditions agrees with most of previous recent studies [52], while the protective effect of moderate alcohol consumption for mental health is still very controversial [53].

Although more research is still needed, other potential covariates that could have an impact on the gender differences in the association between physical inactivity and mental-health conditions among people with vision or hearing impairment are employment or wealth [54], location of households in urban or rural areas [55], quality of sleep [56], degree of adaptation to life with a disability [57], and social support [58].

### 4.3. Implications of Findings

The findings from the present study directly support the implementation of strategies to increase physical-activity levels as a tool for the prevention of mental-health conditions, especially in women with vision loss and in people with vision impairment <65 years. Spanish health authorities should consider these results in order to improve mental health among Spanish women and people who are <65 years old with vision loss. Additionally, it would be recommendable that public health and healthcare practitioners collaborate with sports-science professionals in order to objectively evaluate the levels of physical activity in Spanish women and people who are <65 years old with visual impairment, in an attempt to reduce the prevalence of depression and other mental problems in these population groups.

### 4.4. Strengths and Limitations

The main strengths of our study are the use of data from a large representative survey of the Spanish population, and the use of a validated, reliable and internationally recognized questionnaire to assess physical activity. However, our findings must be interpreted in light of some limitations. First, the study is self-reported, thus potentially introducing recall bias into the findings. Second, as the stem question for mental-health conditions was “have you ever”, it is possible that some participants suffered from mental-health conditions before the existence of physical inactivity. Third, the cross-sectional design did not allow us to establish the direction of the associations. Consequently, future longitudinal studies are required in order to clarify the direction. We recommend that these future studies address the previous limitations and consider the inclusion of other potential covariates that were not measured in this survey, such as the severity of mental-health conditions, employment or wealth, location of households (urban or rural), quality of sleep, degree of adaptation to life with a disability, and social support.

## 5. Conclusions

Our results suggest that physical activity has an important role in the prevention of mental-health conditions, especially in women and people who are <65 years old with vision loss. Therefore, public health and healthcare practitioners should look at appropriate strategies to improve physical inactivity in people with vision loss, especially in women and in those who are <65 years old. These strategies could include practical actions such as the implementation of public, free physical-activity programs for Spanish people with visual impairment and periodic evaluations of their physical activity, in order to achieve and maintain in people with visual impairment the levels of physical activity that are recommended by the World Health Organization.

## Figures and Tables

**Table 1:** Prevalence of physical inactivity in people with vision or hearing impairment, by gender and by mental-health conditions.

Mental-Health Conditions		Vision Impairment Alone (*n* = 2550)						Hearing Impairment Alone (*n* = 1607)					
		Women (*n* = 1540)			Men (*n* = 1010)			Women (*n* = 814)			Men (*n* = 793)		
		Freq (%)	*p*	*d*	Freq (%)	*p*	*d*	Freq (%)	*p*	*d*	Freq (%)	*p*	*d*
Depression	Yes	103 (44.4)	0.009 **	0.167	36 (41.4)	0.158	0.103	38 (55.1)	0.049 *	0.234	20 (43.5)	0.242	0.118
	No	264 (34.9)			227 (33.7)			91 (41.6)			120 (34.7)		
Chronic anxiety	Yes	84 (43.1)	0.056	0.122	36 (45.0)	0.039 *	0.150	31 (49.2)	0.425	0.094	15 (37.5)	0.804	0.025
	No	283 (35.7)			227 (33.4)			98 (43.6)			125 (35.5)		
Other mental problems	Yes	26 (66.7)	<0.001 ***	0.250	9 (56.3)	0.066	0.134	8 (66.7)	0.120	0.184	4 (36.4)	0.964	0.005
	No	341 (35.9)			254 (34.1)			121 (43.8)			136 (35.7)		
Overall		367 (37.1)			263 (34.6)			129 (44.8)			140 (35.7)		

Results presented as frequencies (Valid %). *d* = Cohen’s d. Cohen’s d: small 0.20; medium 0.50; large 0.80. Significant differences between men and women were calculated with chi-square tests: * *p* < 0.05. ** *p* < 0.01. *** *p* < 0.001.

**Table 2:** Association between physical inactivity (exposure) and mental-health conditions (outcome) in people with vision or hearing impairment, by gender, estimated by multivariable logistic regression.

Mental-Health Conditions	Vision Impairment Alone (*n* = 2550)		Hearing Impairment Alone (*n* = 1607)	
	Women (*n* = 1540)	Men (*n* = 1010)	Women (*n* = 814)	Men (*n* = 793)
Depression	1.403 (1.015–1.940) *	1.155 (0.701–1.904)	1.776 (0.960–3.282)	1.170 (0.604–2.265)
Chronic anxiety	1.264 (0.901–1.772)	1.503 (0.908–2.488)	1.225 (0.667–2.250)	0.982 (0.480–2.011)
Other mental problems	2.959 (1.434–6.104) **	3.053 (0.981–9.496)	2.580 (0.520–12.796)	0.503 (0.124–2.049)

Results presented as odds ratio (95% confidence interval). * *p* < 0.05. ** *p* < 0.01. Models adjusted for age, BMI, education level, living as a couple, smoking and alcohol consumption.

## Data Availability

The data that support the findings of this study are available from the corresponding authors upon reasonable request.

## Appendix B

**Table A1:** Association between physical inactivity (exposure), and covariates, with depression (outcome) in people with vision or hearing impairment, by gender, estimated by multivariable logistic regression.

	Vision Impairment Alone (*n* = 2550)		Hearing Impairment Alone (*n* = 1607)	
	Women (*n* = 1540)	Men (*n* = 1010)	Women (*n* = 814)	Men (*n* = 793)
Physical inactivity	1.403 (1.015–1.940) *	1.155 (0.701–1.904)	1.776 (0.960–3.282)	1.170 (0.604–2.265)
Age	1.032 (1.017–1.048) ***	1.023 (0.998–1.049)	1.025 (0.996–1.054)	0.995 (0.963–1.028)
BMI	1.020 (0.989–1.052)	1.054 (1.004–1.107) *	1.054 (0.988–1.125)	1.001 (0.929–1.079)
Primary education	1.291 (0.798–2.087)	1.251 (0.576–2.717)	4.688 (1.427–15.408) *	3.026 (0.961–9.526)
Secondary education	1.128 (0.742–1.714)	1.097 (0.542–2.223)	3.923 (1.213–12.685) *	1.463 (0.462–4.635)
Living as a couple	0.612 (0.447–0.838) **	0.476 (0.291–0.780) **	0.331 (0.178–0.616) ***	0.524 (0.269–1.022)
Current smoker	1.498 (1.013–2.216) *	3.078 (1.528–6.197) **	1.718 (0.782–3.773)	2.027 (0.885–4.640)
Past smoker	1.432 (0.952–2.153)	1.986 (0.947–4.168)	1.471 (0.661–3.272)	1.037 (0.432–2.487)
Alcohol	0.563 (0.404–0.786) ***	0.396 (0.235–0.668) ***	0.956 (0.502–1.821)	0.469 (0.237–0.928) *

Results presented as odds ratio (95% confidence interval). * *p* < 0.05. ** *p* < 0.01. *** *p* < 0.001. Models adjusted for age, BMI, education level, living as a couple, smoking and alcohol consumption.

**Table A2:** Association between physical inactivity (exposure), and covariates, with chronic anxiety (outcome) in people with vision or hearing impairment, by gender, estimated by multivariable logistic regression.

	Vision Impairment Alone (*n* = 2550)		Hearing Impairment Alone (*n* = 1607)	
	Women (*n* = 1540)	Men (*n* = 1010)	Women (*n* = 814)	Men (*n* = 793)
Physical inactivity	1.264 (0.901–1.772)	1.503 (0.908–2.488)	1.225 (0.667–2.250)	0.982 (0.480–2.011)
Age	1.006 (0.991–1.021)	1.017 (0.992–1.042)	1.016 (0.987–1.045)	0.966 (0.936–0.998) *
BMI	1.026 (0.994–1.060)	1.002 (0.949–1.058)	1.067 (1.001–1.138) *	0.980 (0.903–1.064)
Primary education	1.460 (0.880–2.421)	1.812 (0.804–4.082)	0.996 (0.387–2.563)	2.831 (0.862–9.303)
Secondary education	1.232 (0.800–1.898)	1.529 (0.726–3.223)	1.388 (0.576–3.344)	1.695 (0.532–5.396)
Living as a couple	0.723 (0.521–1.003)	0.586 (0.354–0.971) *	0.701 (0.382–1.289)	0.690 (0.338–1.410)
Current smoker	2.099 (1.410–3.125) ***	1.785 (0.885–3.599)	1.651 (0.781–3.490)	1.170 (0.501–2.734)
Past smoker	1.573 (1.021–2.425) *	2.422 (1.194–4.911) *	0.776 (0.338–1.783)	0.935 (0.385–2.269)
Alcohol	0.754 (0.532–1.068)	0.495 (0.289–0.845) *	1.413 (0.741–2.693)	0.472 (0.231–0.966) *

Results presented as odds ratio (95% confidence interval). * *p* < 0.05. *** *p* < 0.001. Models adjusted for age, BMI, education level, living as a couple, smoking and alcohol consumption.

**Table A3:** Association between physical inactivity (exposure), and covariates, with other mental problems (outcome) in people with vision or hearing impairment, by gender, estimated by multivariable logistic regression.

	Vision Impairment Alone (*n* = 2550)		Hearing Impairment Alone (*n* = 1607)	
	Women (*n* = 1540)	Men (*n* = 1010)	Women (*n* = 814)	Men (*n* = 793)
Physical inactivity	2.959 (1.434–6.104) **	3.053 (0.981–9.496)	2.580 (0.520–12.796)	0.503 (0.124–2.049)
Age	0.980 (0.953–1.008)	0.979 (0.935–1.024)	0.968 (0.924–1.015)	0.965 (0.910–1.023)
BMI	1.070 (1.007–1.136) *	1.017 (0.903–1.145)	0.958 (0.791–1.160)	1.160 (0.999–1.347)
Primary education	1.631 (0.580–4.582)	0.934 (0.197–4.420)	2.117 (0.200–22.431)	>1000 (>1000–>1000) ***
Secondary education	0.589 (0.204–1.701)	0.277 (0.052–1.458)	1.847 (0.163–20.951)	>1000 (>1000–>1000)
Living as a couple	0.315 (0.152–0.656) **	0.293 (0.085–1.010)	0.383 (0.079–1.851)	0.105 (0.021–0.530) **
Current smoker	1.327 (0.585–3.009)	2.003 (0.442–9.072)	4277^−9^ (4277^−9^–4277^−9^)	1.509 (0.339–6.713)
Past smoker	0.618 (0.200–1.907)	2.332 (0.482–11.286)	0.747 (0.079–7.043)	0.386 (0.054–2.743)
Alcohol	0.344 (0.155–0.763) **	0.138 (0.041–0.469) **	0.140 (0.015–1.277)	0.177 (0.045–0.701) *

Results presented as odds ratio (95% confidence interval). * *p* < 0.05. ** *p* < 0.01. *** *p* < 0.001. Models adjusted for age, BMI, education level, living as a couple, smoking and alcohol consumption.

**Table A4:** Association between physical inactivity (exposure) and mental-health conditions (outcome) in people with vision or hearing impairment, by gender and age, estimated by multivariable logistic regression.

Mental-Health Conditions	Age (Years)	Vision Impairment Alone (*n* = 2550)		Hearing Impairment Alone (*n* = 1607)	
		Women (*n* = 1540)	Men (*n* = 1010)	Women (*n* = 814)	Men (*n* = 793)
Depression	<65	1.429 (1.010–2.021) *	1.151 (0.679–1.953)	1.765 (0.848–3.672)	1.055 (0.478–2.327)
	≥65	1.107 (0.440–2.785)	1.109 (0.189–6.504)	2.264 (0.591–8.672)	1.413 (0.338–5.913)
Chronic anxiety	<65	1.364 (0.955–1.947)	1.753 (1.037–2.963) *	1.131 (0.549–2.331)	0.994 (0.455–2.173)
	≥65	0.594 (0.186–1.895)	0.196 (0.017–2.254)	2.052 (0.565–7.455)	0.623 (0.088–4.416)
Other mental problems	<65	3.101 (1.416–6.791) **	4.287 (1.267–14.510) *	4.020 (0.581–27.831)	0.359 (0.054–2.384)
	≥65	1.837 (0.225–14.977)	2881^−43^ (0-.)	2.020 (0.076–53.823)	1811^−16^ (0-.)

Results presented as odds ratio (95% confidence interval). * *p* < 0.05. ** *p* < 0.01. Models adjusted for BMI, education level, living as a couple, smoking and alcohol consumption.
